# Integrating Brain-Computer Interface Systems into Occupational Therapy for Enhanced Independence of Stroke Patients: An Observational Study

**DOI:** 10.3390/medicina61050932

**Published:** 2025-05-21

**Authors:** Erika Endzelytė, Daiva Petruševičienė, Raimondas Kubilius, Sigitas Mingaila, Jolita Rapolienė, Inesa Rimdeikienė

**Affiliations:** Department of Rehabilitation, Faculty of Nursing, Medical Academy, Lithuanian University of Health Sciences, 44307 Kaunas, Lithuania; daiva.petruseviciene@lsmu.lt (D.P.); raimondas.kubilius@lsmu.lt (R.K.); sigitas.mingaila@lsmu.lt (S.M.); jolita.rapoliene@lsmu.lt (J.R.); inesa.rimdeikiene@lsmu.lt (I.R.)

**Keywords:** neurorehabilitation, motor imagery, sensorimotor recovery, activities of daily living, neuroplasticity

## Abstract

*Background and Objectives*: Brain-computer interface (BCI) technology is revolutionizing stroke rehabilitation by offering innovative neuroengineering solutions to address neurological deficits. By bypassing peripheral nerves and muscles, BCIs enable individuals with severe motor impairments to communicate their intentions directly through control signals derived from brain activity, opening new pathways for recovery and improving the quality of life. The aim of this study was to explore the beneficial effects of BCI system-based interventions on upper limb motor function and performance of activities of daily living (ADL) in stroke patients. We hypothesized that integrating BCI into occupational therapy would result in measurable improvements in hand strength, dexterity, independence in daily activities, and cognitive function compared to baseline. *Materials and Methods*: An observational study was conducted on 56 patients with subacute stroke. All patients received standard medical care and rehabilitation for 54 days, as part of the comprehensive treatment protocol. Patients underwent BCI training 2–3 times a week instead of some occupational therapy sessions, with each patient completing 15 sessions of BCI-based recoveriX treatment during rehabilitation. The occupational therapy program included bilateral exercises, grip-strengthening activities, fine motor/coordination tasks, tactile discrimination exercises, proprioceptive training, and mirror therapy to enhance motor recovery through visual feedback. Participants received ADL-related training aimed at improving their functional independence in everyday activities. Routine occupational therapy was provided five times a week for 50 min per session. Upper extremity function was evaluated using the Box and Block Test (BBT), Nine-Hole Peg Test (9HPT), and dynamometry to assess gross manual dexterity, fine motor skills, and grip strength. Independence in daily living was assessed using the Functional Independence Measure (FIM). *Results*: Statistically significant improvements were observed across all the outcome measures (*p* < 0.001). The strength of the stroke-affected hand improved from 5.0 kg to 6.7 kg, and that of the unaffected hand improved from 29.7 kg to 40.0 kg. Functional independence increased notably, with the FIM scores rising from 43.0 to 83.5. Cognitive function also improved, with MMSE scores increasing from 22.0 to 26.0. The effect sizes ranged from moderate to large, indicating clinically meaningful benefits. *Conclusions*: This study suggests that BCI-based occupational therapy interventions effectively improve upper extremity motor function and daily functions and have a positive impact on the cognition of patients with subacute stroke.

## 1. Introduction

Approximately 80% of stroke survivors experience some degree of motor impairment in their upper limbs [1], which poses major challenges in regaining independence and re-engaging in ADL.

Occupational therapy is central to the post-stroke recovery process, offering a multidisciplinary approach aimed at restoring functional independence and facilitating reintegration into daily life. Traditional occupational therapy interventions focus on motor and cognitive rehabilitation using evidence-based techniques to address impairments and promote adaptive strategies. However, the complexity and variety of stroke-related deficits demand innovative solutions to achieve optimal therapeutic outcomes [2]. Many stroke survivors experience persistent upper limb motor deficits despite intensive traditional therapy, and a significant proportion remain dependent on daily activities even after standard rehabilitation. These challenges underscore the need for novel interventions that enhance recovery through mechanisms like neuroplasticity [3].

In recent years, brain-computer interface (BCI) systems have emerged as a promising advancement in stroke rehabilitation. These systems use advances in neuroscience and engineering to establish direct communication pathways between the brain and external devices, thereby enabling real-time control and feedback. This technology can bypass impaired motor and sensory pathways, providing new opportunities to complement traditional occupational therapy approaches and enable stroke survivors to regain the functions they have lost [4].

Recent evidence supports the growing efficacy of BCI-based interventions for stroke rehabilitation. For instance, a 2025 meta-analysis confirmed that BCI-assisted therapy significantly improves upper limb motor function and daily living activities in patients with subacute stroke, demonstrating good safety profiles [5]. Additionally, BCI-controlled functional electrical stimulation (BCI has shown positive effects on motor recovery in both subacute and chronic stroke phases, with evidence supporting its immediate benefits [6]. These findings highlight the increasing potential of BCI technologies to enhance neuroplasticity and improve functional outcomes after a stroke.

Research has demonstrated that comprehensive rehabilitation, including BCI training, can enhance upper limb motor function more effectively than routine training in patients with subacute stroke [7]. Despite ongoing research and the need for larger-scale studies, current evidence suggests that combining BCI systems with conventional therapy can substantially improve upper limb motor function and ADL performance [8]. Therefore, a comprehensive and individualized approach to rehabilitation that incorporates early BCI interventions alongside traditional OT is essential for optimizing outcomes and promoting meaningful recovery in patients with stroke. The aim of this study was to investigate the beneficial effects of BCI-based interventions on upper limb motor function and ADL in patients with stroke.

## 2. Materials and Methods

### 2.1. Study Design and Subjects

A retrospective study was conducted to analyze data collected from the medical records of patients who received treatment at the Neurorehabilitation Unit, Clinic of Rehabilitation, Hospital of Lithuanian University of Health Sciences Kauno Klinikos (Kaunas, Lithuania) between February 2021 and March 2024. The study protocol was approved by the Ethics Committee of the Lithuanian University of Health Sciences (approval No. 2024.BEC2-074).

The study group consisted of 56 patients with subacute stroke treated at the Neurorehabilitation Unit, all of whom had experienced their first-ever stroke.

Inclusion Criteria. Patients eligible for study inclusion met the following criteria: (1) first-ever ischemic or hemorrhagic stroke confirmed by CT or MRI scans; (2) hand motor deficit (plegia/hemiparesis), muscle strength < 4 points (as assessed by the Lovett scale); (3) time after a stroke no more than 1 month prior to rehabilitation; and (4) received at least 15 BCI-based RecoveriX treatments during rehabilitation.

Exclusion Criteria. The exclusion criteria were as follows: history of a potential additional brain injury (traumatic brain injury, anoxic brain injury) and serious accompanying diseases (severe cardiovascular conditions, advanced cancer, uncontrolled diabetes, or severe psychiatric disorders); complete aphasia or severe cognitive impairment (Mini-Mental State Examination, MMSE, <11 points); participation in other brain stimulation procedures, such as transcranial magnetic stimulation or deep brain stimulation, during the training period; and insufficient pre- and post-rehabilitation assessment data (hand function, independence, or cognitive function testing).

### 2.2. Comprehensive Rehabilitation

All patients received standard medical care and rehabilitation for 54 days as part of the comprehensive treatment protocol. This included routine occupational therapy, which focused on hand function, and physiotherapy, which primarily addressed lower-limb function, gait, and balance. Additionally, interventions such as massage therapy, electrical stimulation, and intermittent compression were used to promote circulation, reduce muscle tension, and support overall motor recovery in patients. Psychological support and counseling were provided by a psychologist to address emotional and mental health needs, while social work services offered assistance with social and community reintegration. Speech therapy sessions were also conducted to address communication and swallowing difficulties. This multidisciplinary approach aimed to optimize recovery outcomes and enhance the overall well-being of stroke survivors.

Occupational therapy focused on the rehabilitation of arm and hand movements used in ADL. The participants engaged in structured hand-training exercises targeting specific aspects of hand function. The exercises included bilateral exercises, grip-strengthening activities, fine motor coordination tasks, tactile discrimination exercises, and proprioceptive training. Mirror therapy was used to enhance motor recovery through visual feedback. Participants received ADL-related training aimed at improving functional independence in everyday activities. Training sessions focused on practicing tasks relevant to an individual’s daily life, such as grooming, dressing, and feeding. The occupational therapist incorporated adaptive strategies, environmental modifications, and assistive devices as needed to facilitate the successful performance of the task.

Routine occupational therapy was provided 5 times per week for 50 min per session. BCI training was provided every second day as shown in the schedule (Table 1), with 15 sessions per patient in total over the course of the rehabilitation. BCI sessions began one week after the start of the rehabilitation program; therefore, for the first week, patients received only conventional occupational therapy. On days when BCI training was scheduled, it replaced routine occupational therapy sessions to ensure that patients had a balanced treatment schedule.

### 2.3. Functional Evaluation

All functional assessments were performed on the first and second days of admission to the Neurorehabilitation Unit, Clinic of Rehabilitation, Hospital of Lithuanian University of Health Sciences, Kauno Klinikos. The assessments were repeated one day before discharge. Patients’ cognitive function was assessed using the MMSE and the Clock Drawing Test (CDT). Patient independence was assessed using the Functional Independence Measure (FIM). The Box and Blocks Test (BBT) and Nine-Hole Peg Test (9HPT) were used to evaluate hand function. Arm strength was assessed using a digital hand dynamometer.

#### 2.3.1. Functional Independence Measure

The FIM was used to assess the functional status of the participants by evaluating their performance in ADL. This involved scoring 18 items across 6 domains, including self-care, sphincter control, mobility, locomotion, communication, and social cognition, to determine the level of assistance required for each activity. Each item is rated on a 7-point scale: 1, total assistance; 2, maximal assistance; 3, moderate assistance; 4, minimal assistance; 5, supervision; 6, modified independence; and 7, complete independence. The total score of this instrument ranges from 18 to 126, with higher scores indicating greater independence. The FIM is commonly used to assess functional recovery and allows for the precise evaluation of patients with moderate-to-severe stroke [9].

#### 2.3.2. Box and Block Test

The BBT was used to assess the manual dexterity of stroke patients by having them move as many blocks as possible from one compartment to another in 60 s. Both stroke-affected and unaffected hands were evaluated separately to compare the dexterity and functional performance of each hand before and after treatment [10].

#### 2.3.3. Nine-Hole Peg Test

The 9HPT is a standardized assessment tool for measuring finger dexterity and coordination. Participants have to place and remove 9 pegs from a board as quickly as possible, with each hand being tested separately. It provides a reliable measure of fine motor skills for both affected and unaffected hands in stroke patients and is widely regarded as the gold standard for evaluating manual dexterity [11].

#### 2.3.4. Dynamometry

A digital dynamometer was used to measure the grip strength of participants. During dynamometry, all patients were seated with their elbows flexed at 90 degrees and their forearms in a neutral position. Each participant performed the test by squeezing the dynamometer with maximum effort for a few seconds, and this was done separately for both the stroke-affected and unaffected hands to assess and compare their muscle strength. All measurements (patient testing) were performed by an occupational therapist working with the patient in the Neurorehabilitation Unit.

A portable dynamometer is considered a valid instrument for measuring isometric muscular strength; it is easy to use, provides objective measurements, and is sensitive for detecting changes in muscle strength [12].

#### 2.3.5. Mini-Mental State Examination

The MMSE is a widely used test forassessing cognitive function [13]. It involves a series of tasks that evaluate various cognitive domains, including orientation, registration, attention and calculation, recall, and language skills. Participants are rated on a 30-point scale, with higher scores indicating better cognitive function. The test helps identify cognitive impairments and track changes over time.

#### 2.3.6. Clock Drawing Test

The CDT is internationally acknowledged as a neuropsychological screening tool, valued for its strong psychometric properties, including high test-retest reliability [14]. A four-point scoring methodology for the CDT was used, which simplifies the evaluation process by focusing on key aspects of the clock drawing. This method assesses four main components, each worth one point: (1) the presence of a closed circle representing the clock face; (2) the presence of all numbers from one to twelve; (3) correct placement and spacing of the numbers around the clock face; and (4) the presence of clock hands correctly indicating ten past eleven.

### 2.4. BCI Training Procedures

BCI training sessions were designed with clear, simplified instructions and provided with additional support as needed to ensure that patients could participate effectively.

The patients received BCI training for 52 min a day, 2–3 times a week. During these sessions, they were instructed to imagine the movement of their affected and unaffected hands while avoiding blinking, coughing, chewing, and any other head and body movements.

**Figure 1:**
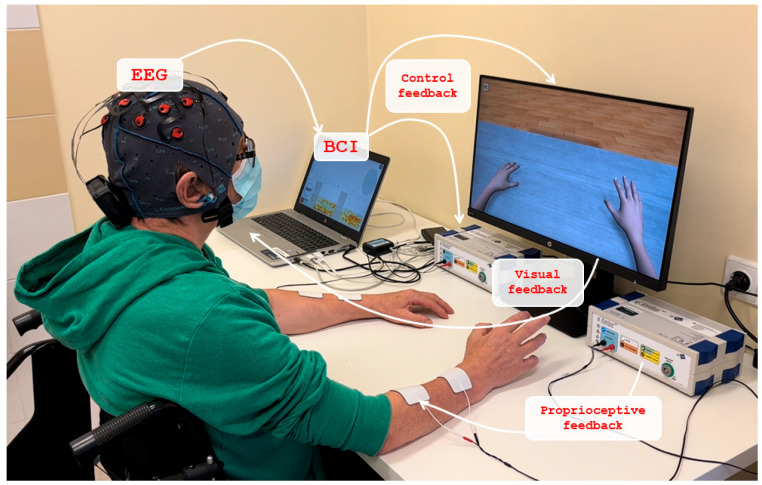
Application of BCI in a clinical setting.

Electroencephalography (EEG) signals were recorded using 16 electrodes, placed on the regions of FC3, FCz, FC4, C5, C3, C1, Cz, C2, C4, C6, CP3, CP1, CPz, CP2, CP4, Pz positions, based on the international 10–10 system and then amplified (recoveriX PRO, g.tec medical engineering GmbH, Schiedlberg, Austria) and computer processed. A video was displayed on screen to guide the patients through each training task (Figure 1).

The patient is seated and wears an EEG cap fitted with electrodes. After the signal, the patient attempts to imagine the movement of his/her hand. As soon as the BCI system detects hand movement, the patient receives visual feedback through a virtual onscreen avatar that reflects the imagined hand movements. Simultaneously, tactile feedback is provided through electrical muscle stimulation. To deliver this feedback, stimulation electrodes are placed on both the affected and unaffected forearms, targeting the extensor digitorum communis (EDC) muscle. Before each training session, the stimulation parameters are calibrated to the patient’s current condition. The stimulation parameters (intensity, frequency, and duration) were individually calibrated for each patient to induce visible and functional dorsiflexion of the affected wrist. The default stimulation frequency was 50 Hz, and the pulse width was set to 300 µs for all patients. The current amplitude was individually adjusted to the maximum level tolerated without discomfort. For safety, the system automatically pauses the stimulation after 15 s of inactivity. Each session consisted of three exercises. The first exercise was performed in calibration mode, and by default, it switched to training mode for the remaining two exercises. Each session was repeated 240 times.

Each movement started with an attention sound, instructing the patient to concentrate on the upcoming task. Two seconds later, a command to imagine the dorsiflexion of either the left or right wrist appeared. The command was given in a pseudo-random order, of which the patient was unaware. The patient tried to imagine the hand movement and had to continue until he/she heard the command to relax.

No active lifting of the arm or any other attempt was required to perform the movement. On the computer screen, the patient saw a virtual image of the hand performing the wrist extension movement of the corresponding hand. Therefore, in this BCI technology scenario, the patient received two types of feedback: visual feedback from observing the virtual hand moving on the computer screen and proprioceptive feedback from the muscle contraction induced by the electro stimulation system. Furthermore, the recoveriX system ensures individual adaptation through a closed-loop feedback mechanism based on real-time EEG analysis. Visual and proprioceptive feedback is delivered only when motor imagery is correctly detected, which encourages accurate engagement and reinforces neuroplasticity. This selective reinforcement not only tailors therapy to the patient’s current cognitive state but also increases their motivation and compliance. By monitoring brain activation in real-time, the system dynamically adjusts feedback delivery, making each session functionally adapted to the patient’s mental and motor performance levels.

### 2.5. Statistical Data Analysis

Statistical analyses were performed using the Statistical Package for the Social Sciences (SPSS) version 29.0, with a significance level set at *p* < 0.05. The distribution of the data was assessed using the Shapiro-Wilk test or Kolmogorov–Smirnov tests. The demographic characteristics of the patients are expressed as means with standard deviations (SD). Other data were non-normally distributed and are expressed as medians with interquartile ranges (IQR) to reflect the central tendency and dispersion. Nonparametric tests were used for the subsequent analyses. The Mann−Whitney U test was used to compare two independent groups. For the correlation analysis, Spearman’s rank correlation coefficient was used to assess the relationships between variables. Effect sizes were calculated using the rank-biserial correlation (r), which is appropriate for nonparametric data analyzed with the Wilcoxon signed-rank test. Values of r of around 0.1, 0.3, and 0.5 were interpreted as small, medium, and large effects, respectively.

## 3. Results

Ischemic stroke was diagnosed in 31 participants and hemorrhagic stroke in 25 participants (Table 2). The participants’ ages ranged from 28 to 89 years. The mean age was 61.47 years (SD, 12.65) for males and 63.05 years (SD, 11.64) for females.

### 3.1. Changes in Hand Function

At the beginning of rehabilitation, 23 patients had complete paralysis (plegia) of the stroke-affected hand and could not perform dynamometry, whereas 33 were diagnosed with paresis. Patients with hand paresis (n = 33) at the beginning of rehabilitation had a median hand strength of 5.00 (IQR, 0.00–9.50) kg. When assessing the strength of the unaffected hand of all participants (n = 56), the median strength was 29.65 (IQR, 21.00–40.00) kg. Table 3 shows the outcomes of patients’ hand function, cognitive function, and independence in daily living.

At the end of rehabilitation, 11 subjects still had plegia in the stroke-affected hand. In patients with hand paresis (n = 45), the median strength of the stroke-affected hand was 6.65 (IQR, 1.75–18.75) kg and that of the stroke-unaffected hand was 37.20 (IQR, 25.00–43.00) kg. The change in strength in both hands was statistically significant (*p* < 0.001).

The rehabilitation program resulted in statistically and clinically significant improvements in hand strength, dexterity, and fine motor control in both stroke-affected and unaffected hands.

Hand dexterity, as measured by the BBT, also showed marked improvement. At the beginning of rehabilitation, 32 patients with stroke-affected hands were unable to move even a single block per min. At the end of rehabilitation, the number of patients with such injuries decreased to 11.

Fine motor control, assessed using the 9HPT, also demonstrated similar improvements. Initially, 37 patients with stroke-affected hands could not complete the test within 5 min. By the end of the program, the number of patients with such conditions decreased to 25. Those who were able to complete the test showed a median improvement of 133 s.

The rehabilitation program was associated with statistically significant improvements in hand strength, dexterity, and fine motor control, as shown by the outcome measures.

### 3.2. Changes in Activities of Daily Living

At the beginning of rehabilitation, patients had a median FIM score of 43 (IQR, 36.25–50.75). The assessment of patients’ independence showed that they needed total assistance in 10 of the 18 ADL areas. Initially, patients had the most difficulty with bathing, dressing the upper and lower bodies, toileting, bladder and bowel management, performing transfers, walking, and climbing stairs.

**Figure 2:**
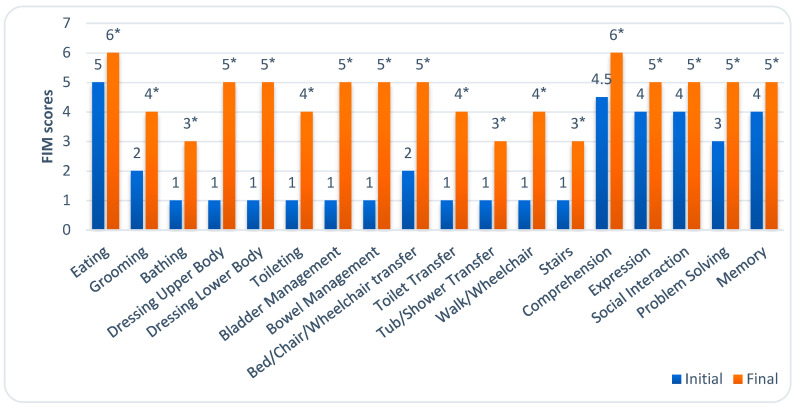
Changes in different ADL areas after occupational therapy with BCI technology were applied. Values are medians. * *p* < 0.001, compared to the FIM score before rehabilitation.

At the end of rehabilitation, patients’ independence statistically significantly improved, with a median FIM score of 83.50 (IQR, 70.00–96.70). Patients still required moderate assistance with bathing, toileting, performing transfers to the tub or shower, and climbing stairs. However, most activities could only be performed under supervision. Figure 2 illustrates the level of dependence in all 18 ADL areas before and after rehabilitation.

Patients with paresis or plegia at the beginning of rehabilitation showed statistically significant improvements in FIM scores after rehabilitation (Table 4). The Wilcoxon signed-rank test showed a significant improvement in FIM scores from the initial to final evaluation in the plegia (Z = −4.20, *p* < 0.001) and paresis groups (Z = −5.02, *p* < 0.001). The Mann−Whitney U test for two independent groups revealed that patients with plegia had significantly lower functional independence scores both at the beginning (U = 185.50, *p* < 0.001) and at the end (U = 170.0, *p* < 0.001) of rehabilitation compared with those with paresis after stroke.

### 3.3. Changes in Cognitive Function

At the beginning of rehabilitation, 39% of patients had moderate cognitive impairment, 41% had mild impairment, and 20% had normal cognitive function, with a median MMSE score of 22.00 (IQR, 18.25–26). By the end of rehabilitation, significant improvements were observed: only 4% had moderate impairment, 48% had mild impairment, and 48% had normal function, with a median MMSE score of 26.00 (IQR, 25.00–29.00) (*p* < 0.001). Similarly, CDT scores improved from a median of 3.00 (IQR, –1.00; 4.00) to 4.00 (IQR, 3.00–4.00) (*p* < 0.001).

The moderate positive correlation between the FIM and MMSE (ρ = 0.405, *p* = 0.002) and CDT (ρ = 0.396, *p* = 0.003) scores at the beginning of rehabilitation showed that patients with higher cognitive function had higher functional independence in ADL. At the end of rehabilitation, patients’ independence was still positively correlated with cognitive status as measured by the MMSE (ρ = 0.392, *p* = 0.003), but there was no correlation between the FIM and CDT scores (ρ = 0.222, *p* = 0.099).

## 4. Discussion

This observational study analyzed the data of 56 patients with subacute stroke. All participants underwent occupational therapy enhanced with BCI-based sessions aimed at improving hand function during rehabilitation.

The application of BCI systems in stroke rehabilitation is an evolving field. At present, BCI systems are more frequently employed during the chronic phase of stroke recovery than in the subacute phase. Nevertheless, starting rehabilitation early after a stroke could be essential to fully utilize the therapeutic potential [15], as it involves brain neuroplasticity. Neuroplasticity encompasses a dynamic process of cellular, molecular, and synaptic changes that enable the brain to adapt, learn, and repair itself despite neurological damage caused by a stroke. This remarkable adaptability offers a promising foundation for creating innovative approaches aimed at enhancing the recovery of lost functions [3].

Our findings are consistent with those of previous studies showing that BCI-based rehabilitation improves upper limb function more than conventional therapy alone [7,16].

A meta-analysis by Cervera et al. reported that BCI-based neurorehabilitation for upper limb motor function showed a medium-to-large effect size, improving the scores of the Fugl-Meyer Assessment for the upper extremity (FMA-UE) more effectively than conventional therapies. Additionally, the analysis highlighted evidence that BCI could trigger functional and structural neuroplasticity at a subtle level, with some of these changes linked to better motor recovery [17]. This further underscores the growing evidence supporting the efficacy of BCI in facilitating recovery in patients with stroke.

Many patients with stroke miss the optimal recovery window and enter the chronic stage. In these cases, innovative technologies like BCI could offer renewed hope for functional improvement. Studies combining BCI with functional electrical stimulation have also reported greater functional gains than standard therapy [18]. These findings suggest that BCI, particularly when combined with other modalities like FES, can provide significant benefits beyond the subacute phase.

Among the various methods of upper limb rehabilitation, those that offer sensory feedback (e.g., visual feedback through mirror therapy) or somatosensory stimulation (e.g., transcutaneous electrical stimulation) seem to be beneficial [19]. By enhancing sensory feedback, these approaches are more effective in influencing neuroplasticity and facilitating recovery.

The addition of different types of feedback to rehabilitation could help improve neurophysiological changes and boost motor performance. This combined approach using enhanced feedback appears to be more effective for rehabilitation, particularly in stroke survivors [15]. Research shows that BCI combined with mental practice (MP) and occupational therapy is a promising tool for promoting recovery of the upper limb and functional independence in subacute post-stroke survivors [20].

In our study, patients received two forms of feedback: visual feedback by watching the virtual hand moving on the computer screen and proprioceptive feedback from muscle contractions triggered by the electrostimulation system. This dual feedback approach is crucial as it reinforces the brain’s ability to reorganize itself through multiple sensory channels. The selection of wrist extension movement training in BCI protocols is driven by its feasibility, accessibility, functional relevance, muscle activation patterns, and versatility, making it a practical and effective choice for enabling BCI control in rehabilitation and assistive technology settings [21].

Wrist extension is a fundamental movement involved in many ADL, such as reaching, grasping, and manipulating objects. By training individuals to generate wrist extension commands using BCI technology, they can potentially regain control over essential functional movements and improve their independence in performing daily tasks. Moreover, wrist extension movements are relatively straightforward to detect and classify using EEG, a common modality for BCI systems, making it an optimal target for rehabilitation [21].

A secondary analysis of combined data (n = 223) in a study by Ghaziani et al. revealed that shoulder abduction, finger extension, and elbow extension were strong predictors of using the arm for basic ADL. In contrast, wrist extension and supination were linked to the use of the arm for routine ADL 6 months after stroke [22]. Similarly, Nijland et al. found that the ability to extend fingers and abduct the shoulder within 2 days post-stroke predicted a 98% chance of scoring at least 10 points on the Action Research Arm Test(ARAT) at 6 months post-stroke [23]. In a large group of stroke patients, shoulder abduction, finger extension, and elbow extension predicted basic ADL ability, while wrist extension and supination were associated with routine ADL use at 6 months. These findings highlight the critical role of specific motor functions in the long-term recovery process. Patients with a less favorable prognosis may require longer rehabilitation, and repeated assessments can help refine long-term recovery estimates [22].

Sebastián-Romagosa et al. conducted a study with 51 stroke patients who had upper extremity hemiparesis: 45 patients were in the chronic phase (88.2%) and 6, in the subacute phase (11.8%). All participants completed 25 sessions of motor imagery-based BCI training, with assessments performed before and after training. The results showed significant improvements: the motor function of the paretic arm assessed using the FMA-UE increased by 4.68 points (*p* < 0.001), while wrist and finger spasticity assessed using the Modified Ashworth Scale decreased by 0.72 and 0.63 points, respectively (both *p* < 0.001). Importantly, these functional gains persisted for 6 months post therapy. The study concluded that motor imagery-based BCI training effectively enhanced long-lasting functional improvements in upper extremity motor function, which was explained by improved neuroplasticity [24].

The results of our study indicated that the majority of patients with subacute stroke experienced significant improvements in strength, dexterity, and fine motor skills in both affected and unaffected hands by the end of rehabilitation. In addition to statistically significant improvements, effect size analysis using rank-biserial correlation showed large effects across most outcomes (r ≈ 0.65–0.87), further supporting the clinical relevance of BCI-based interventions in subacute stroke rehabilitation. Initially, 23 of the 56 patients had complete paralysis (plegia), preventing the use of objective measurement tools such as the BBT, 9HPT, and dynamometry. At the end of rehabilitation, 11 patients still exhibited plegia in their affected hands. Similar challenges in hand function assessment and partial improvements have also been reported in other BCI studies with subacute stroke patients [25].

Hand function plays a crucial role in many ADL. After a stroke, impairments in hand function, such as weakness, spasticity, decreased dexterity, and sensory deficits, can significantly impact a person’s ability to perform various tasks. Following a stroke, most patients experience impairment of arm function, which persists in up to 60–67% of individuals, according to different authors [14,26]. The common goal of BCI-based therapy is to improve functional independence. Monitoring changes in ADL performance could offer a way to evaluate how well a BCI supports this objective [27].

Our study found that patients showed significant improvements in functional independence by the end of rehabilitation. However, those with plegia had lower FIM scores than those with paresis. Analysis of individual ADL domains assessed by the FIM test revealed the most notable improvements in independence in upper and lower body dressing, toileting, sphincter control, transfers, and mobility.

A systematic review by Wondergem et al., identified nine factors linked to a decline in ADL after stroke. Moderate evidence supports a relationship between dependence on ADL and impaired leg motor function. Limited evidence was found for factors such as insurance status, living alone, age 80 or older, inactivity, impaired cognitive function, depression, and fatigue. These findings suggest that impaired ADL and motor function may lead to a more inactive lifestyle, further complicating post-stroke recovery [28]. Most recovery in ADL usually occurs within the first 6 weeks after a stroke and is closely linked to the initial severity of the stroke. Additionally, dependency in personal ADL during the first 2 days after a stroke is thought to predict dependency at 3 and 12 months post-stroke [29]. In our study, we found a moderately strong association between patients’ independence in ADL and cognitive impairment. Other studies have reported similar results, indicating that cognitive impairment is a significant factor explaining ADL dependency 3 months post-stroke [30]. Another systematic review and meta-analysis found a significant medium association between cognition and basic ADL (eating, dressing, and toileting) and instrumental ADL (housework and social interactions) [31].

A significant obstacle in employing EEG-based motor imagery-BCI in stroke patients is the cognitive demand for sustained attention and the risk of inducing fatigue during training. These factors may make it unsuitable for many stroke patients and reduce its effectiveness [32]. Post-stroke cognitive impairment, a prevalent symptom following stroke, has been reported to affect 53.4% of patients in recent studies. Most therapeutic approaches, including BCI-based motor rehabilitation, require a minimum level of cognitive ability for patients to comprehend and follow the instructions essential for the rehabilitation process [33].

In a neurorehabilitation department, a considerable number of patients experience ongoing cognitive impairment that limits their capacity to engage in BCI-based training. These impairments often result in difficulties in comprehending instructions, particularly in visualizing arm movements as required by the procedure. Additionally, many patients struggle to maintain concentration for the duration of nearly hour-long sessions, with the repetitive nature of the tasks often leading to drowsiness and incomplete procedures. However, for some patients, BCI interventions can be considered later in their rehabilitation process, once an improvement in cognitive function to a moderate impairment level by the MMSE has been achieved. Studies do not specify what level of cognitive function is optimal, but Cantillo-Negrete et al., in their comprehensive guide to BCI-based stroke neurorehabilitation interventions, emphasized the importance of assessing cognitive functions to ensure that impairments are not too severe [34].

Game mechanics can be applied to help users sustain their interest in training while concealing the actual performance of the BCI system [35]. By integrating game elements, the monotony of repetitive tasks can be reduced, potentially enhancing patient engagement and improving the outcomes. A pilot study evaluating the impact of gamification on the efficiency of rehabilitation found that incorporating game features such as scoring and time stimuli did not negatively affect performance. Additionally, participants reported that game elements did not hinder their ability to create mental imagery and made the process feel less tedious [36].

Preliminary results of post-stroke cognitive rehabilitation using BCI indicate promising potential for addressing non-motor deficits, including cognitive and emotional impairments. Furthermore, previous research has highlighted the complex relationship between motor, cognitive, and emotional functions, which can significantly impact overall post-stroke rehabilitation outcomes [37]. This suggests evidence that a more integrated approach to BCI-based rehabilitation could provide broad benefits across various functional domains.

In recent years, BCI has been used to address various neurological disorders. Additionally, researchers have implemented diverse BCI approaches to enhance post-stroke cognitive impairment, which plays a crucial role in managing these impairments and preventing vascular dementia and other related conditions [33]. In our study, all patients who received BCI-based treatment demonstrated a statistically significant improvement in cognitive function. Although some patients were not able to begin BCI treatment on the first day of rehabilitation, all participants ultimately completed 15 BCI-based treatment sessions.

The optimal number of sessions required for most patients to achieve minimal clinically significant recovery remains a subject of debate. However, studies suggest that the number of therapy sessions, ranging from 10 to 60, may be required to reach this rehabilitation goal [34]. Other research, however, proposes that in stroke rehabilitation using BCI, the frequency of therapy sessions might be less critical than the overall dose and intensity of the therapy [38].

BCI systems offer unique opportunities to enhance stroke rehabilitation in the subacute and chronic stages by providing real-time feedback, facilitating neuroplasticity, and enabling individuals to regain lost motor and cognitive functions. By customizing these systems to align with each patient’s specific needs and capabilities, they can play a vital role in facilitating continued recovery beyond the acute phase of stroke.

However, commercially available BCI devices are often too expensive for broader use, mainly due to their technical complexity and costly materials. Their bulky design also limits their application in laboratory or clinical settings. To improve accessibility, especially in resource-limited environments, future systems should be portable and cost-effective [39].

While these considerations highlight the potential of BCI systems in stroke rehabilitation, it is important to address the limitations of this study that may influence the interpretation and application of its results. One of the limitations of this study is that some participants were unable to perform all the tests needed to assess hand function due to persistent hand muscle weakness. Moreover, a significant limitation is the challenge in distinguishing the effects of BCI therapy from the benefits of concurrent occupational therapy, physical therapy, and other rehabilitation treatments that patients underwent during the study period. Despite this, training sessions with feedback contributed to the recovery of patients’ hand function, demonstrating the positive effects of the BCI. However, the lack of a control group in our study somewhat restricts our ability to draw definitive conclusions regarding the efficacy of BCI therapy alone. Another limitation of this study is the lack of long-term follow-up data after the intervention period. Due to the retrospective nature of the study, it was not possible to assess the sustainability of the improvements beyond discharge. Future prospective studies should address this by incorporating follow-up evaluations at later stages, such as 6- or 12-months post-rehabilitation.

## 5. Conclusions

This study demonstrated a statistically significant and clinically meaningful improvement in motor function, cognitive function, and independence in the daily life of patients when BCI-enhanced occupational therapy sessions were applied during the subacute stroke period.

However, further research is needed to evaluate the long-term effectiveness and sustainability of such interventions, beyond the initial rehabilitation phase. In addition, the generalizability of the results is limited by the relatively small sample size of a single-center study.

## Figures and Tables

**Table 1:** An example of the recoveriX application schedule for each patient.

Monday	Tuesday	Wednesday	Thursday	Friday	Saturday	Sunday
recoveriX	conventional OT	recoveriX	conventional OT	recoveriX	-	-
conventional OT	recoveriX	conventional OT	recoveriX	conventional OT	-	-

**Table 2:** Demographic and clinical characteristics of the study participants (n = 56).

Characteristic	Value
Gender, n (%)	
Male	34 (60.7)
Female	22 (39.3)
Age, mean (SD), years	
Male	61.47 (12.65)
Female	63.05 (11.64)
Lesion side, n (%)	
Right	28 (50)
Left	28 (50)
Stroke type, n (%)	
Ischemic	31 (55.4)
Hemorrhagic	25 (44.6)
Time after stroke, mean (SD), days	12 (4)
Muscular weakness, n (%)	
Plegia	23 (41.1)
Paresis	33 (58.9)

**Table 3:** Assessment of motor, cognitive, and daily functions at the beginning and end of rehabilitation.

Test	N	Evaluation	Median (IQR)	Z-Value	*p*-Value	Effect Size (r)
MMSE, score	56	Initial	22.00 (18.25–26.00)	–6.47 ^†^	0.001	−0.864
	56	Final	26.00 (25.00–29.00)			
CDT, score	56	Initial	3.00 (1.00–4.00)	–4.85 ^†^	0.001	−0.648
	56	Final	4.00 (3.00–4.00)			
FIM, total score	56	Initial	43.00 (36.25–50.75)	–6.51 ^†^	0.001	−0.87
	56	Final	83.50 (70.00–96.75)			
Dynamometry, kg (not affected hand)	56	Initial	29.65 (21.00–40.00)	–4.89 ^†^	0.001	−0.653
	56	Final	37.20 (25.00–43.00)			
Dynamometry, kg (affected hand)	33	Initial	5.00 (0.00–9.50)	–5.84 ^†^	0.001	−0.871
	45	Final	6.65 (1.75–18.75)			
BBT, blocks/min (not affected hand)	56	Initial	45.00 (36.00–54.00)	–6.22 ^†^	0.001	−0.831
	56	Final	57.00 (47.00–66.00)			
BBT, blocks/min (affected hand)	24	Initial	0.00 (0.00–11.50)	–5.51 ^†^	0.001	−0.822
	45	Final	15.00 (0.50–34.50)			
9HPT, s (not affected hand)	56	Initial	29.00 (23.00–35.00)	–5.32 ^‡^	0.001	−0.711
	56	Final	21.00 (18.00–28.00)			
9HPT, s (affected hand)	19	Initial	180.00 (98.00–245.00)	–4.11 ^‡^	0.001	−0.737
	31	Final	47.00 (32.00–86.00)			

MMSE—Mini-Mental State Examination; CDT—Clock Drawing Test; FIM—Functional Independence Measure; BBT—Box and Block Test; 9HPT—Nine-Hole Peg Test; Z-value—Wilcoxon Signed Ranks Test, ^†^ based on negative ranks, ^‡^ based on positive ranks; *p*-value indicates the statistical significance of the difference between initial and final evaluations; r—rank-biserial correlation; effect size for Wilcoxon Signed Ranks Test, values above 0.5 are considered large effects.

**Table 4:** Within- and between-group comparisons of FIM scores.

Hand Impairment	FIM	N	Median (IQR)	Z-Value	U-Value	*p*-Value
Within-Group Comparisons						
Plegia	Initial	23	37 (26–44)	–4.20 ^a^	N/A	0.001
	Final	23	69 (58–87)			
Paresis	Initial	33	45 (41.5–54)	–5.02 ^a^	N/A	0.001
	Final	33	86 (78–102)			
Between-Group Comparisons (plegia vs. paresis)						
Plegia	Initial	23	37 (26–44)	–3.24 ^b^	185.50	0.001
Paresis	Initial	33	45 (41.5–54)			
Plegia	Final	23	69 (58–87)	–3.49 ^b^	170.00	0.001
Paresis	Final	33	86 (78–102)			

FIM, Functional Independence Measure; Z-value, ^a^ Wilcoxon Signed Ranks Test; ^b^ Mann-Whitney U test.

## Data Availability

Requests for access to the datasets underlying this study should be directed to the corresponding author for consideration in accordance with the institutional or ethical guidelines governing data sharing and participant confidentiality.

## Abbreviations

The following abbreviations are used in this manuscript:

ADL	Activities of Daily Living
BCI	Brain-Computer Interface
FES	Functional Electrical Stimulation
EDC	Extensor Digitorum Communis
BBT	Box and Block Test
9HPT	Nine-Hole Peg Test
FIM	Functional Independence Measure
MMSE	Mini-Mental State Examination
CDT	Clock Drawing Test
EEG	Electroencephalography
OT	Occupational Therapy
EDC	Extensor Digitorum Ciqrommunis
SPSS	Statistical Package for the Social Sciences
IQR	Interquartile Range
ARAT	Action Research Arm Test
FMA-UE	Fugl-Meyer Assessment of Upper Extremity
